# Genomic Landscape and Regulation of RNA Editing in Pekin Ducks Susceptible to Duck Hepatitis A Virus Genotype 3 Infection

**DOI:** 10.3390/ijms251910413

**Published:** 2024-09-27

**Authors:** Haonao Zhao, Zifang Wu, Zezhong Wang, Jinlong Ru, Shuaiqin Wang, Yang Li, Shuisheng Hou, Yunsheng Zhang, Xia Wang

**Affiliations:** 1 College of Animal Science and Technology, Northwest A&F University, Yangling 712100, China; zhaohaonan66@nwafu.edu.cn (H.Z.); zifang_wu@126.com (Z.W.); wangzz2024@126.com (Z.W.); jinlong.ru@gmail.com (J.R.); yangli2001@nwsuaf.edu.cn (Y.L.); 2 Institute of Animal Sciences, Chinese Academy of Agricultural Sciences, Beijing 100193, China; wangshq86@126.com (S.W.); houss@263.net (S.H.)

**Keywords:** RNA editing, DHAV-3, Pekin ducks, susceptibility, miRNA

## Abstract

RNA editing is increasingly recognized as a post-transcriptional modification that directly affects viral infection by regulating RNA stability and recoding proteins. the duck hepatitis A virus genotype 3 (DHAV-3) infection is seriously detrimental to the Asian duck industry. However, the landscape and roles of RNA editing in the susceptibility and resistance of Pekin ducks to DHAV-3 remain unclear. Here, we profiled dynamic RNA editing events in liver tissue and investigated their potential functions during DHAV-3 infection in Pekin ducks. We identified 11,067 informative RNA editing sites in liver tissue from DHAV-3-susceptible and -resistant ducklings at three time points during virus infection. Differential RNA editing sites (DRESs) between S and R ducks were dynamically changed during infection, which were enriched in genes associated with vesicle-mediated transport and immune-related pathways. Moreover, we predicted and experimentally verified that RNA editing events in 3′-UTR could result in loss or gain of miRNA–mRNA interactions, thereby changing the expression of target genes. We also found a few DRESs in coding sequences (CDSs) that altered the amino acid sequences of several proteins that were vital for viral infection. Taken together, these data suggest that dynamic RNA editing has significant potential to tune physiological processes in response to virus infection in Pekin ducks, thus contributing to host differential susceptibility to DHAV-3.

## 1. Introduction

As a widespread post-transcriptional mechanism, RNA editing can modify RNA sequences by inserting/deleting or substituting site-specific bases to make them different from genomic DNA templates [1,2,3]. Being mediated by endogenous deaminases, RNA editing can be effective against specific viruses [4]. Among the types of post-transcriptional nucleotide modifications, adenosine to inosine (A-to-I) RNA editing catalyzed by adenosine deaminase acting on RNA (ADAR) enzymes and cytidine to uridine (C-to-U) editing catalyzed by the apolipoprotein B mRNA editing catalytic polypeptide-like (APOBEC) enzymes are two current focuses in the field [5,6,7]. In the course of viral infections, ADARs can modulate cellular responses by acting directly through hypermutation of viral RNA or indirectly by editing host transcripts [5,8,9]. While APOBECs utilize C-to-U hypermutation or non-enzymatic pathways that interfere with reverse transcription to target viral genomes, typically DNA intermediates [10,11,12,13]. Since the translational machinery recognizes inosine (I) as guanosine (G) and uracil (U) as thymine (T), RNA editing in the coding regions of the genome may result in amino acid substitutions that alter the function of the protein [2].

Rapid advances in high-throughput transcriptome sequencing technology have made it possible to globally screen for various types of RNA editing events. However, when identifying RNA editing events, some false positive signals caused by single-nucleotide polymorphisms (SNPs) and comparison errors are also recognized [12], making establishing reliable genome-wide mapping of RNA editing sites difficult for most organisms. The current best solution is to sequence both genomic DNA and mRNA from the same sample to eliminate most false positive signals [2].

As a species characterized by fast growth rate, high feed conversion rate, high lean meat rate, and a low skin-to-fat ratio, Pekin duck is an ideal raw material and major source that may be used to meet the requirements of poultry meat production [13,14]. However, Pekin duck farming has been plagued by duck viral hepatitis (DVH), a highly fatal infectious viral disease that usually occurs in ducklings less than 3 weeks old, thus affecting both the production and economic viability of farms [15]. The ability to control this disease is critical for the sustainability of the duck industry. Over the past few years, DHAV-3 has become the most prevalent pathogen of DVH in the Asian duck industry [16,17,18]. To mitigate the negative effects of this disease, our group started a selective breeding program to genetically improve the resistance of ducks to DHAV-3 about 10 years ago. As of now, a resistant Pekin duck flock (Z8) has been obtained with a mortality rate of 10%, which is much lower than that of a susceptible Pekin duck flock (Z7) (90%) [19,20]. The mechanism of DHAV-3 infection in Pekin ducks has been explored from the perspectives of gene expression, lipid expression, and ncRNA expression [21,22,23,24]. Nevertheless, due to the complexity of virus–host interactions, the potential regulatory function of many other aspects of the mechanism, such as RNA editing, in the interaction between DHAV-3 and Pekin ducks remains unclear.

In this study, we systematically identified and categorized a landscape of RNA editing events in susceptible and resistant Pekin ducks at 0 h, 12 h, and 24 h after DHAV-3 infection based on direct comparison of omics data from transcriptome sequencing and genome resequencing. Furthermore, this study explored the potential regulatory functions of RNA editing in Pekin ducks’ responses to DHAV-3 infection. These findings suggest a potential role for RNA editing in regulating miRNA–mRNA interactions and protein functions in Pekin ducks during DHAV-3 infection. Ultimately, these findings hypothesized that RNA editing plays a crucial role in the differential responses of Pekin ducks to DHAV-3 infection, which will enhance OUR understanding of the pathogenic mechanisms of DHAV-3 and the varied responses of Pekin ducks.

## 2. Results

### 2.1. Genome Resequencing and Transcriptome Data Evaluation

After performing high-throughput sequencing and quality control, the average total number of clean reads obtained from the raw transcriptome reads was about 110 M for each sample, corresponding to more than 10× coverage of resequencing genome data. More than 90% transcriptome and genome reads were successfully mapped to the reference duck genome.

### 2.2. RNA Editing Profiles in S and R Ducks during Infection

**Figure 1:**
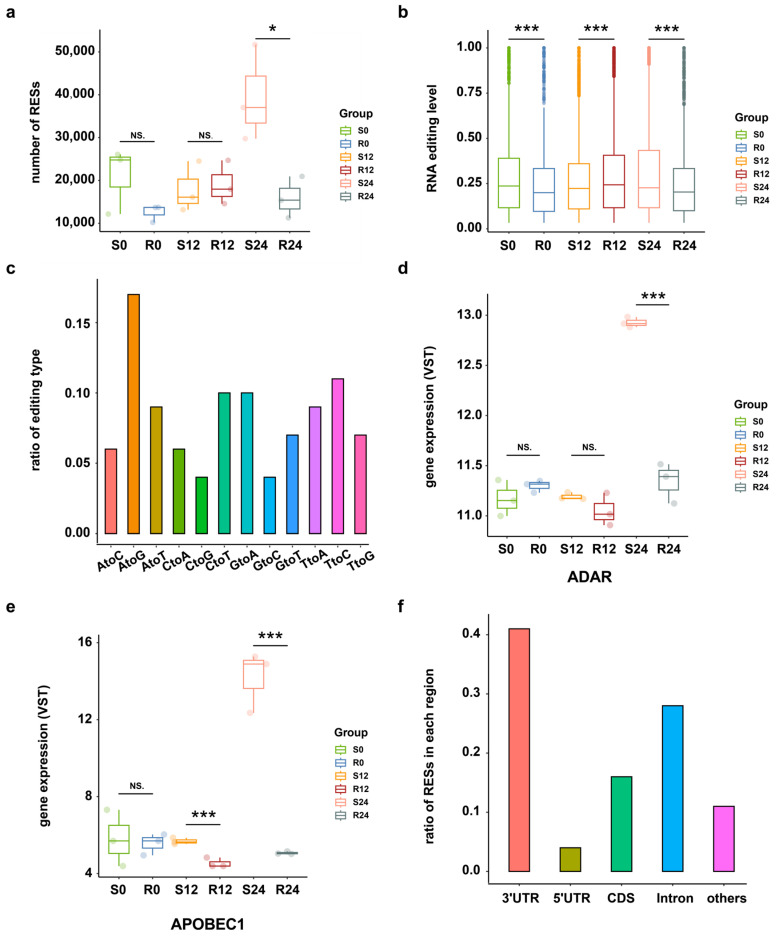
RNA editing events overview. (**a**) Numbers of RNA editing sites in S and R ducks at 0 hpi, 12 hpi, and 24 hpi. * *p* < 0.05; NS. *p* > 0.05. (**b**) Editing level distribution of all RESs across the DHAV-3 infection in S and R ducks, as calculated by the average editing level of three biological replicates of each group. *** *p* < 0.001. (**c**) Statistics of RNA editing types in all samples. (**d**) The expression level of ADAR across the DHAV-3 infection in S and R ducks. *** *p* < 0.001; NS. *p* > 0.05. (**e**) Expression level of APOBEC1 across the DHAV-3 infection in S and R ducks. *** *p* < 0.001; NS. *p* > 0.05. (**f**) Distribution of RESs in different regions.

To investigate the RNA editing events across DHAV-3 infection of S and R ducks, the transcriptome sequencing data and matched DNA sequencing data of 18 samples were utilized from three time points after infection (0 hpi, 12 hpi, and 24 hpi). A total of 11,067 putative RESs were identified from these samples, with more RNA editing sites (RESs) occurring in S ducks at 24 hpi than in the other groups (Figure 1a). RESs were more strongly edited in S ducks than R ducks at 24 hpi (Figure 1b). Among those putative RESs, A-to-G had the highest ratio of 17%, following by T-to-C (11%) and C-to-T (10%) (Figure 1c). Because the T-to-C edits may result from A-to-I editing on overlapping antisense RNAs by ADAR1 [25], the top three editing events detected may reflect RNA editing by members of the ADAR and AID/APOBEC family of deaminases, in which the expression of ADAR1 and APOBEC1 is higher in S24 compared to the other conditions (Figure 1d,e) (see Supplementary Materials Figure S1). According to the genome distribution analysis, 41%, 16%, 28%, and 4% of RESs were located in 3′-UTR, CDS, Intron, and 5′-UTR regions, respectively (Figure 1f).

### 2.3. Differentially Expressed RESs (DRESs) between S and R Ducks during Infection

**Figure 2:**
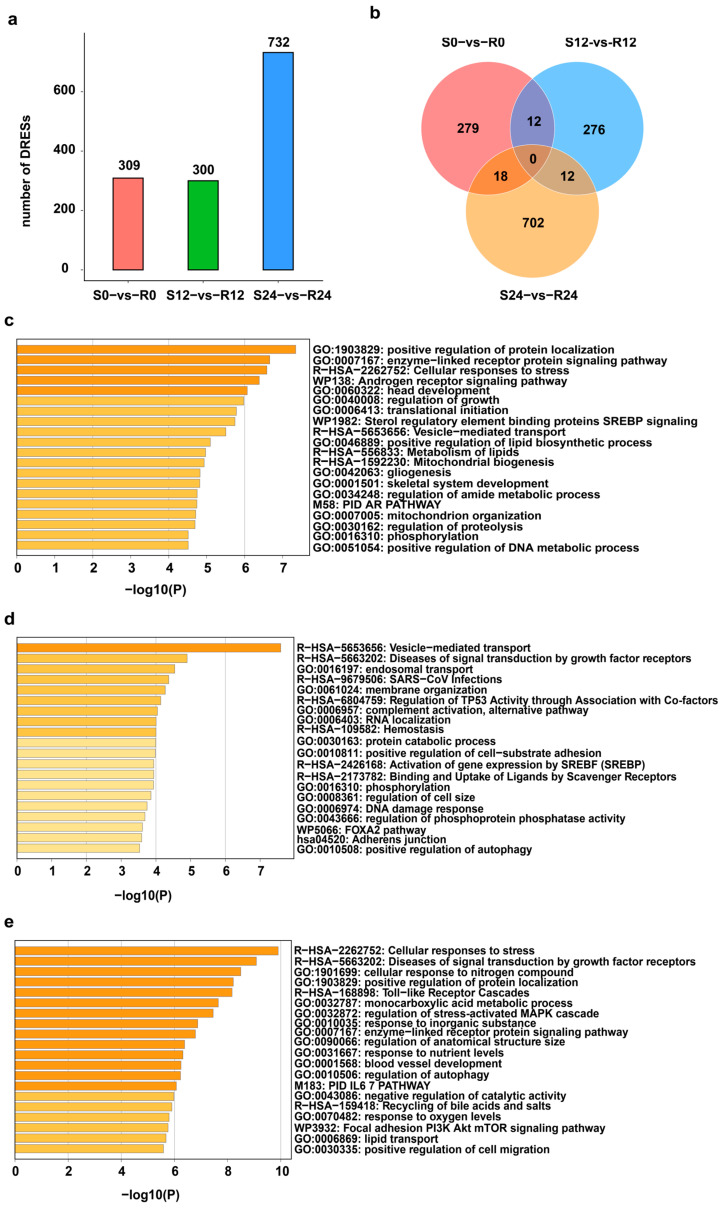
Profiles of DRESs during DHAV-3 infection. (**a**) Number of DRESs in S and R ducks during DHAV-3 infection. (**b**) Venn diagram of DRESs in S0-vs-R0, S12-vs-R12, and S24-vs-R24. (**c**–**e**) Significant enriched terms of genes containing DRESs at 0 hpi, 12 hpi, and 24 hpi.

A total of 309, 300 and 732 differentially expressed RESs (DRESs) were identified in S0-vs-R0, S12-vs-R12, and S24-vs-R24, respectively (Figure 2a). The number of DRESs increased across the DHAV-3 infection, with the most DRESs appearing in S24-vs-R24, suggesting that the effect of DHAV-3 infection on editing events becomes stronger during the 12–24 hpi period (Figure 2a). However, only a few DRESs identified in S-vs-R ducks were shared between any two time points, and no DRESs were shared among three time points (Figure 2b), suggesting dynamic editing patterns during infection with DHAV-3.

To elucidate the biological function of the genes covering DRESs across DHAV-3 infection, enrichment analysis were performed at 0 hpi, 12 hpi and 24 hpi. As shown in Figure 2c, the genes covering DRESs at 0 hpi were significantly enriched in processes such as “positive regulation of protein localization” and the “enzyme-linked receptor protein signaling pathway”. At 12 hpi, genes covering DRESs were significantly enriched in “vesicle-mediated transport”, “SARS-CoV infections” and so on (Figure 2d). Moreover, genes covering DRESs were related to the functions at 24 hpi, including “Cellular responses to stress”, “Toll-like receptor cascades”, and “positive regulation of cell migration”. (Figure 2e). These results suggest that DRESs in these genes associated with vesicle-mediated transport and immune-related pathways may affect DHAV-3 infection.

### 2.4. DRESs in 3′ UTR Alter mRNA-miRNA Regulation during Infection

**Figure 3:**
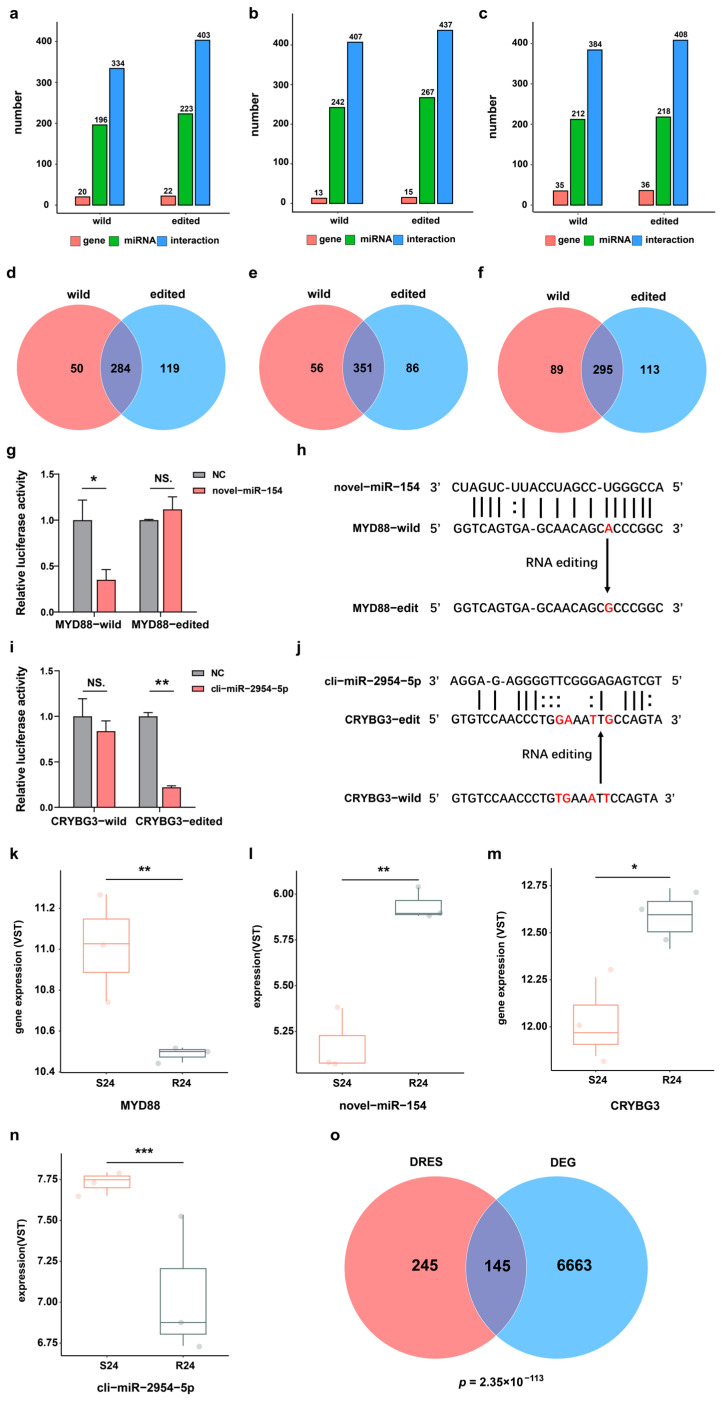
DRESs in 3′-UTR affect miRNA binding sequences and target gene expression. (**a**–**c**) Statistics of miRNAs and their predicted targets for wildtype and RNA-edited transcripts at 0 hpi, 12 hpi, and 24 hpi. (**d**–**f**) Venn diagrams about the number of miRNA–mRNA interactions predicted based on wildtype and edited transcripts at 0 hpi, 12 hpi, and 24 hpi. (**g**) Relative luciferase activity in HEK293T cells carrying wildtype or edited *MYD88* 3′-UTR regulated by novel-miR-154. NS. *p* > 0.05; * *p* < 0.05. (**h**) Schematics of an RNA editing event disrupting the interaction between novel-miR-154 and *MYD88*. The RNA editing site is colored in red. (**i**) The relative luciferase activity in HEK293T cells carrying wildtype or edited *CRYBG3* 3′-UTR affected by cli-miR-2954-5p. NS. *p* > 0.05; ** *p* < 0.01. (**j**) Schematics of RNA editing events receiving the interaction between cli-miR-2954-5p and *CRYBG3*. RNA editing sites are shown in red color. (**k**–**n**) The expression level of *MYD88*, novel-miR-154**,** *CRYBG3*, and cli-miR-2954-5p in S and R ducks at 24 hpi. * *p* < 0.05; ** *p* < 0.01; *** *p* < 0.001. (**o**) Venn diagram of genes harboring DRESs and DEGs in S24-vs-R24. The *p*-value was calculated based on Fisher’s exact test.

The editing events of miRNA binding sites in 3′-UTR of mRNA may perturb post-transcriptional regulation by miRNA or mRNA binding proteins, thereby altering the expression of corresponding genes. Hence, all DRESs in the 3′-UTR were first selected between the S and R groups during infection. To explore whether the DRESs in 3′-UTR can affect miRNAs binding to target genes, 539 unique and highly expressed mature miRNAs (452 known and 87 novel) previously identified in duck were utilized [26], and their potential binding affinity to wildtype and corresponding RNA-edited mRNA transcripts was then predicted. It was found that 196 miRNAs had 334 interactions with 20 wildtype genes, while 223 miRNAs had 403 interactions with 22 RNA-edited genes at 0 hpi (Figure 3a) (see Supplementary Materials Figure S2a,b). Comparing interactions of miRNAs with wildtype targets and RNA-edited targets, 119 new miRNA–mRNA binding relationships were gained, and 50 miRNA–mRNA binding relationships were lost (Figure 3d). At 12 hpi, 242 miRNAs interacted with 13 wildtype targets, and 267 miRNAs interacted with 15 RNA-edited targets (Figure 3b) (see Supplementary Materials Figure S2c,d). As a result, a total of 86 gained interactions and 56 lost interactions were identified (Figure 3e). At 24 hpi, 384 interactions were predicted among 212 miRNAs and 35 wildtype targets, while 218 miRNAs and 36 RNA-edited targets had 408 interactions (Figure 3c) (see Supplementary Materials Figure S2e,f), resulting in 456 gained interactions and 197 lost interactions (Figure 3f). These results also illustrated that the occurrence of dynamic RNA editing events in the 3′-UTR regions may interrupt regulation of gene expression by miRNAs.

To further verify the effects of RNA editing events on miRNA–target binding relationships, we selected one lost and one gained miRNA–mRNA interaction (lost: *MYD88* and novel-miR-154, gained: *CRYBG3* and cli-miR-2954-5p) caused by DRESs. The luciferase activity was downregulated sharply when novel-miR-154 and Luc-MYD88-WT were co-transfected into the cells compared with the transfection of Luc-MYD88-WT (NC) (Figure 3g), indicating that the novel-miR-154 can bind to wildtype *MYD88* 3′-UTR and repress its expression. However, the luciferase activity did not change when novel-miR-154 and Luc-MYD88-ED were co-transfected into the cells compared to transfection of Luc-MYD88-ED (NC) (Figure 3g), suggesting that the novel-miR-154 cannot bind to RNA edited *MYD88* 3′-UTR (NC_051773.1:159267277, A-to-G) to repress its expression (Figure 3h). Consistently, *MYD88* mRNA expression was upregulated in S24 compared with R24 (Figure 3k). Meanwhile, novel-miR-154 expression was also downregulated in S24 vs. R24, suggesting that alteration of *MYD88* mRNA abundance may attributed to both novel-miR-154 expression change and DRES in binding site of novel-miR-154 (Figure 3l). In contrast to *MYD88*, a dual luciferase assay showed that cli-miR-2954-5p can repress expression of *CRYBG3* by targeting mutated *CRYBG3* 3′-UTR (NC_051772.1:96023793, T-to-G; NC_051772.1:96023794, G-to-A; NC_051772.1:96023797, A-to-T; NC_051772.1:96023799, T-to-G) but not wildtype *CRYBG3* 3′-UTR (Figure 3i,j). In line with this observation, *CRYBG3* expression was downregulated in S24 compared with R24, which may be due to cli-miR-2954-5p expression change and gained an interaction between cli-miR-2954-5p and *CRYBG3* (Figure 3m,n). Moreover, genes containing DRESs in their 3′-UTR were significantly enriched with differentially expressed genes (DEGs) between S and R ducks at 24 hpi (Figure 3o). These results suggested that RNA editing events possibly regulated gene expression via miRNA modulation mechanisms.

### 2.5. DRESs in CDS Influence Protein Functions during DHAV-3 Infection

**Figure 4:**
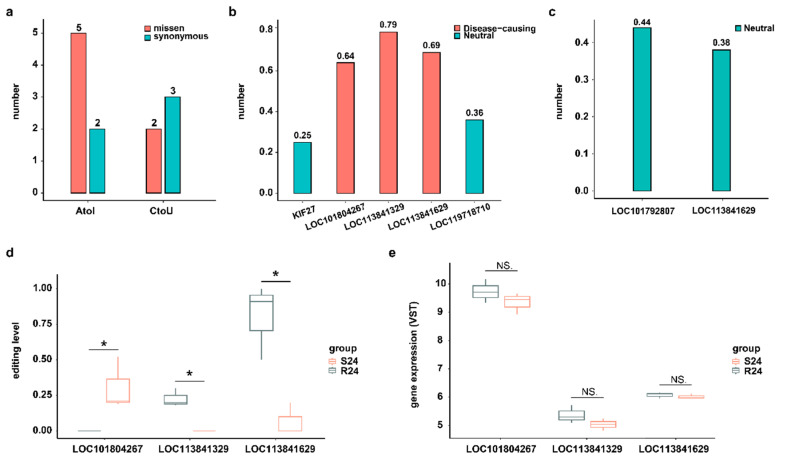
DRESs in CDS influence protein functions. (**a**) A-to-I and C-to-U DRESs caused missense and synonymous variants between S and R ducks at 24 hpi in CDS region. (**b**) Predicted variant effects of A-to-I DRESs at 24 hpi. The horizontal coordinates represent the corresponding genes covering the DRESs. (**c**) Predicted variant effects of C-to-U DRESs at 24 hpi. The horizontal coordinates represent the corresponding genes covering the DRESs. (**d**) The editing level of A-to-I DRESs caused a missense variant at 24 hpi. The horizontal coordinates represent the corresponding genes covering the DRESs. (**e**) Expression of genes covering A-to-I DRESs which caused missense variant at 24 hpi. NS. *p* > 0.05; * *p* < 0.05.

Although most RNA editing events occur in the noncoding regions of the genome, editing events that do take place in coding sites may alter the protein sequence and thus potentially change its function [27]. In this study, we focused on two well-known types of DRESs (A-to-I and C-to-U) located on the CDS region at 24 hpi. A total of 7 A-to-I and 5 C-to-U DRESs were identified at 24 hpi, of which 5 A-to-I and 2 C-to-U DRESs resulted in missense mutations on LOC113841629 (KIF27-like), LOC101804267 (CYP2W1-like), LOC119718710, LOC113841329 (ATAD2-like), KIF27, and LOC101792807 (ZDHHC20-like) (Figure 4a). Then, the functional consequences of DRESs causing missense variants were predicted using a machine learning-based pathogenicity method, MutPred2. Only three A-to-I type missense variants with scores > 0.6 were predicted to be pathogenic, while all others were non-pathogenic (Figure 4b,c). Notably, a DRES at site 45 converting Y (Tyrosine, Tyr) to S (Serine, Ser) on LOC113841329 (ATAD2-like) was predicted to be pathogenic with score of 0.79 (Figure 4b), which intersects both the predicted interaction and the high conservation, suggesting the biological importance of this site. Although the editing levels of these three pathogenic mutations were different between S24 and R24, their mRNA expression levels were similar in two conditions (Figure 4d,e).

**Figure 5:**
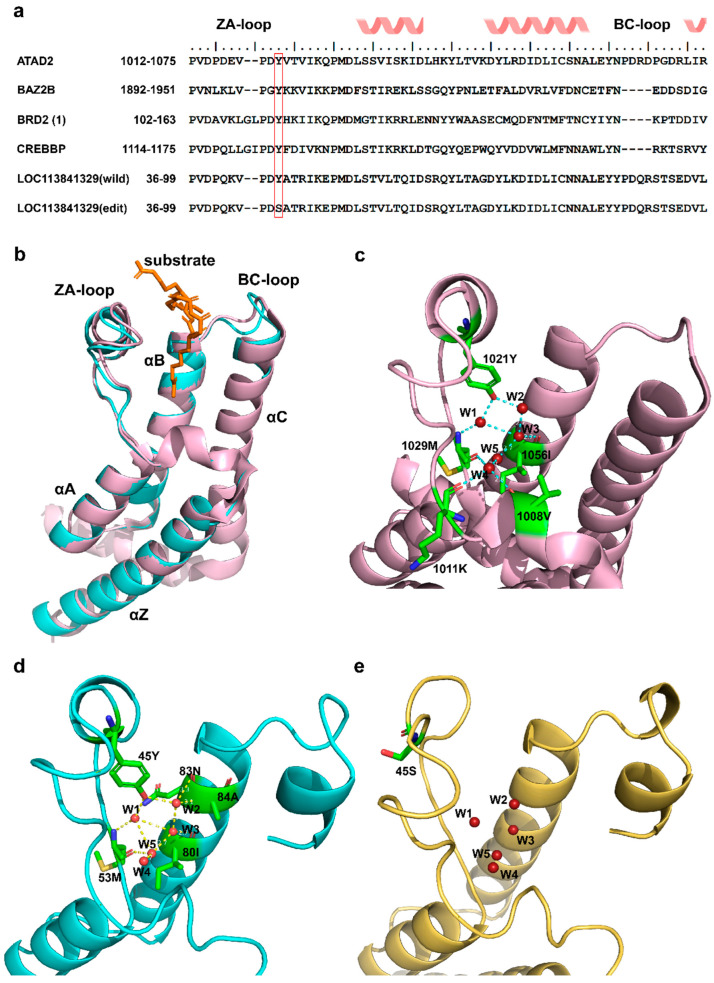
Three-dimensional structures of three bromodomains. (**a**) Sequence alignment of six bromodomain pockets for ATAD2 (PDB: 7m98), BAZ2B (PDB: 3G0L), BRD2(1) (PDB: 1X0J), CREBBP (PDB: 3DWY), wildtype LOC113841329, and edited LOC113841329. The red frame shows the conserved Y residue through the entire bromodomain family. (**b**) Overlay of 3D structures of two bromodomains. Pink: ATAD2 (PDB: 7m98); cyan: wildtype LOC113841329. (**c**) Close-up of binding sites with structural waters shown in ATAD2 (PDB: 7m98). (**d**) Close-up of binding sites with structural waters shown in wildtype LOC113841329. (**e**) Close-up of binding sites with structural waters shown in edited LOC113841329.

LOC113841329 is predicted to be a homologue of the ATAD2 bromodomain, which is a small protein module that recognizes acetylated lysine on histones [28], thereby connecting to many diseases, including cancer, inflammation, and viral infection [29,30]. ATAD2 is therefore generally referred to as an oncogene. Thus, inhibition of LOC113841329/ATAD2 may suppress progression of tumor or virus infection. Notably, previous study found that diverse members of the bromodomain family shared a highly similar three-dimensional structure and a highly conserved KAc (lysine acetylation) pocket, although they had different sequences, as shown in Figure 5a,b [28]. 45Y, which is located in the ZA loop of wildtype LOC113841329 (corresponding to 1021Y in ATAD2 (PDB:7m98)), is one of the highly conserved residues forming the KAc pocket (Figure 5b,c). After superimposition of LOC113841329 to the crystal structure of ATAD2 (7m98), it was found that the ZA loop was stabilized by the conserved 45Y, which anchored structured water molecules at the base of the binding pocket, while the Y45S mutation disrupted the hydrogen bonds of 45Y with structured water molecules, thus affecting the dynamics of ZA loop and the conformation of hydrophobic acetyl–lysine binding site in edited LOC113841329 (Figure 5e). Therefore, the RNA editing at site 45 in LOC113841329 in R24 probably impaired the oncogenic activity of this gene, although there was no significant difference in the expression levels of this gene between S24 and R24 (Figure 4d,e).

## 3. Discussion

DHAV-3 infection poses a threat to the Pekin duck industry in Asia [13,31]. Investigating the DHAV-3–host interaction is useful in understanding the pathology of DHAV-3 infection in Pekin ducks. This study provides a first description of the comprehensive set of RNA editing sites in Pekin ducks during DHAV-3 infection, comprising 11,067 unique and high-quality RESs identified in liver whole-transcriptome samples. Consistent with previous studies [6,32,33], A-to-I RNA editing was the main type among the 12 identified types in this study. As the genome distribution analysis showed, 41% of RESs were located in the 3′-UTR region, followed by 16% in the CDS region. This is in line with previous studies which have found that most known editing sites are located in non-coding regions, such as UTRs and introns of genes [6,34,35,36]. Since some RESs located in the CDS region can influence protein structure and thereby affect biological function, these sites are also significant [6,37].

As expected, the significant increase in the editing catalyzing genes APOBEC and ADAR in S24 compared to R24 corresponded to a greater number of DRESs observed in S24 versus R24. Moreover, those DRESs were mainly enriched in the biological functions associated with cell activation, immune, and metabolic processes, suggesting that they might be involved in virus infection. Notably, we found a significant difference in RNA editing in “Toll-like Receptor Cascades” between two groups, suggesting RNA modifications affect stimulation of TLRs in the innate immune system. Previous studies have indicated that miRNAs can regulate mRNA translation and stability by binding to miRNA targets in 3′-UTR [38,39,40]. The occurrence of RNA editing events in the 3′-UTR region of mRNAs may result in the loss of originally matching miRNA target sites or the creation of new miRNA targets [39,41,42], thereby regulating corresponding gene expression [36,42]. Consistently, this study also observed that RNA editing of NC_051773.1:159267277 (A-to-G) in *MYD88* (a downstream signaling molecule of TLRs) in S24 vs. R24 eliminated the binding of novel-miR-154, leading to the upregulation of *MYD88* in S24. Meanwhile, the four differentially consecutive editing sites (NC_051772.1:96023793, T-to-G; NC_051772.1:96023794, G-to-A; NC_051772.1:96023797, A-to-T; NC_051772.1:96023799, T-to-G) in *CRYBG3* obtained a new binding site of cli-miR-2954-5p, resulting in the downregulation of this gene in S24 vs. R24. Recent studies have demonstrated that *MYD88* upregulation during many viral infections impaired host antiviral type I IFN response, and its dysregulation/over-activation contribute to exacerbated inflammatory response, the so-called cytokine storm, in many virus diseases [43,44,45]. As an important protein structure regulator gene, *CRYBG3* is likely to be involved in the regulation of platelet size and volume, which play an important role in blood coagulation and inflammatory response during virus infection [46]. Thus, the dysregulation of RNA editing sites in 3′-UTR leads to abnormal expression of *MYD88* and *CRYBG3*, promoting DHAV-3 infection in S ducks at 24 hpi.

The RNA editing events in the CDS region may change amino acid, thereby affecting protein functions [27]. In this study, seven unique nonsynonymous DRESs (five A-to-I and two C-to-U) were identified at 24 hpi. Moreover, three substitutions in A-to-I type edited protein were predicted to be pathogenic. Notably, LOC113841329 (ATAD2-like) is potentially an oncoprotein. A DRES at conserved site 45 from Y to S in LOC113841329 in R24 destabilized the ZA loop, thereby affecting substrate binding. This alteration could attenuate the oncogenic function of LOC113841329 in R24, but not in S24. The roles of these mutations are context-dependent, which has also been observed in other genes; for example, some mutations can promote EZH2 activity, which is consistent with the oncogenic role of EZH2, while others inactivate the function of EZH2, suggesting that it can also work as a tumor suppressor [47]. Therefore, RNA editing sites in CDS led to changes in amino acid, which affected the corresponding protein functions and consequently altered DHAV-3 infection in Pekin ducks at 24 hpi.

Overall, these findings indicate that RNA editing plays an important role in DHAV-3 infection. Similar roles of RNA editing have been observed in other viral infection such as SARS-CoV-2. For example, theMAVS gene, which functions as a “switch” in the immune signal transduction against most RNA viruses, displayed dramatically altered RNA editing across different SARS-CoV-2-infected tissues [48,49]. Nevertheless, further functional studies in future to validate the RNA editing changes identified in this study could provide a deeper understanding pathology of DHAV-3 infection. On the other hand, this study focused on the RNA editing in ducks, but not on RNA modifications in viruses. It has been found that the RNA modifications in viruses may not only affect host recognition of virus RNA but also modulate host sensitivity to innate immune effector molecules [50]. Therefore, detecting RNA modifications in viruses would be valuable for future investigation of DHAV-3 infection in susceptible and resistant Pekin ducks.

## 4. Materials and Methods

### 4.1. Ethics Statement

The experiments were approved by the Animal Welfare and Ethics Committee of the Institute of Animal Sciences (IAS), Chinese Academy of Agricultural Sciences (IAS20160401, CAAS, Beijing, China).

### 4.2. Virus and Animals

The highly virulent 112,803 strain of DHAV-3 used in this study was propagated in 9-day-old embryonic Pekin duck eggs and stored at −80 °C. Virus titer was determined as 10^6.83^ 50% egg lethal dose (ELD50) per 0.2 mL.

A flock of Pekin ducks susceptible to DHAV-3, designated S, and a resistant line of Pekin ducks, designated R, were kept for this study on a Pekin duck breeding farm within the Institute of Animal Sciences, Chinese Academy of Agricultural Sciences, Beijing, China.

### 4.3. Samples Collection

Nine ducks from the S flock and nine ducks from the R flock were randomly selected in this study. At 7 days of age, each individual was inoculated intramuscularly with 0.5 mL (2.5 × 10^6.83^ ELD50/0.2 mL) of DHAV-3 112803. Three S and three R ducks were euthanized and necropsied at 0, 12, and 24 hpi, respectively, and fresh liver tissue samples were collected from each duck. The S ducks at 0 hpi, 12 hpi and 24 hpi were denoted the S0 group, S12 group, and S24 group, respectively, while the R ducks at these three time points were denoted the R0 group, R12 group, and R24 group, respectively.

### 4.4. Genome and Transcriptome Sequencing

The liver samples were homogenized in 20% PBS, and the supernatant was collected after centrifugation at 12,000× *g* for 15 min at 4 °C. Genomic DNA was extracted using phenol/chloroform. According to the manufacturer’s instructions, two paired-end libraries (300 bp) were constructed using a kit provided by Illumina (Illumina, San Diego, CA, USA), and the whole genome was resequenced using a Hiseq 2500 sequencing platform (Illumina). RNA was extracted from supernatant according to the Trizol method. By using the TruSeq stranded RNA library sample Prep Kit (Illumina, San Diego, CA, USA), the cDNA libraries were constructed, and then the paired-end reads were generated based on a Hiseq 2500 sequencing platform (Illumina, San Diego, CA, USA).

### 4.5. Quality Control and Read Mapping

High-quality clean reads were obtained by removing reads containing adapters or polyN sequences and low-quality reads via fastp software (version 0.23.4). RNA-paired clean reads were aligned and sorted using STAR software (version 2.7.10a), while DNA-paired clean reads were aligned and sorted using bwa software (version 0.7.17) and samtools software (version 1.17), respectively.

### 4.6. RNA Editing Site Detection and Annotation

In order to prevent the detection of SNPs (single-nucleotide polymorphisms) as RNA editing events, the transcriptome sequencing data and matched genome resequencing data were utilized from 18 samples.

RNA editing sites (RESs) were then detected de novo using reditools software (version 2.0) with the default parameters. To reduce errors of identified RESs, only those meeting the following thresholds were retained: a minimum of 3 RNA-seq reads supporting the variation, a minimum read coverage of 10 for RNA-seq, and a minimum RNA editing frequency of 0.1. The RESs were further filtered by excluding the sites in repeat regions based on the RepeatMasker annotations and the DNA mutation variants that were supported by the paired genome resequencing data. We then annotated the ZJU1.0 version duck reference genome and annotation files, the distribution of RESs on the genome, and the RESs on which genes were identified.

### 4.7. Differential RNA Editing Analysis

The RESs supported by at least two samples at each time point were retained, and the editing level of each RES was calculated as the ratio of reads covering the edited base to the total number of reads covering the site. To identify the differential RNA editing sites (DRESs) between R and S groups at each time point, a single-tailed Mann–Whitney test method was employed, with a threshold set at *p* < 0.05. Additionally, a comprehensive analysis of functional and pathway enrichment was conducted to annotate genes with DRESs using Metascape (https://metascape.org, accessed on 5 October 2023).

### 4.8. Analysis of the RNA Editing Effects on miRNA Regulation

To investigate the effect of DRESs in 3′-UTR on miRNA binding, the 3′-UTR sequences were extracted from the reference genome using R package “GenomicRanges” (version 1.56.1) and bedtools software (version 2.30.0) as wildtype sequences. The mutations were then applied according the type of DRESs to obtain the edited 3′-UTR sequences. Mature miRNA sequences in duck were retrieved from previous work [26]. After removing miRNAs with duplicated sequences or low expression, miRNA–mRNA 3′-UTR interactions were predicted using miRanda software (3.3a) with a minimum free energy (MFE) cutoff at −20 kcal/mol in both wildtype and edited scenarios, and then we constructed the interaction networks using Cytoscape software (version 3.9.1). Regarding the wildtype 3′-UTR (miRNA target region) as control, the gain/loss of the miRNA–mRNA interactions caused by DRESs was quantified.

### 4.9. Verification of the RNA Editing Effects on miRNA–mRNA Interactions

One gained and one lost miRNA–mRNA interaction (gained: CRYBG3 and cli-miR-2954-5p, lost: MYD88 and novel-miR-154) caused by DRESs was selected for a dual luciferase reporter assay to verify the RNA editing effects on miRNA–mRNA interactions. Fragments of ~500 bp of CRYBG3 and MYD88 3′-UTRs spanning the wildtype (WT) or edited (ED) miRNA binding sites were cloned into the pGL3-Basic reporter plasmid (Sangon, Shanghai, China). The miRNA sequences were also synthesized by Sangon (Shanghai, China). HEK-293T cells were then co-transfected with Luc-CRYBG3-WT/Luc-CRYBG3-ED and cli-miR-2954-5p mimics/pGL3-Basic/miR-control (RiboBio, Guangzhou, China). Another interaction, MYD88 and novel-miR-154, was co-transfected by the same method. After 48 h of incubation, the activities of firefly and Renilla luciferase were measured using a Dual Luciferase Reporter Assay Kit (UElandy, Suzhou, China).

### 4.10. Analysis of RNA Editing’s Effects on Protein Structures

To investigate the implications of RNA editing for changes in protein functions, two major DRESs of A-to-I editing and C-to-U editing were calculated using a single-tailed Mann–Whitney test with a threshold of *p* < 0.05, and DRESs located in CDS regions at 24 hpi were selected for subsequent analysis. Using bedtools (version 2.30.0), the wildtype transcript sequences covering DRESs were extracted from reference; subsequently, the wildtype transcript sequences were mutated to edited sequences, and both wildtype and edited sequences were translated into corresponding amino acid sequences using the R package “Biostrings” (version 2.72.1). The nonsense mutations of these sequences were identified, and their 3D protein structures were then predicted by Alphafold2 (version 2.3.0).

### 4.11. Prediction of Functional Effects of RNA Editing

The various effects of amino acid substitutions on the RNA-edited proteins were predicted using MutPred2 (version 2.0). The prediction score ranged from 0 to 1, representing low to high probability of pathogenicity [51].

## 5. Conclusions

This study collected transcriptome and DNA-seq data from liver tissue of susceptible and resistant Pekin ducks and comprehensively detected RNA editing events during DHAV-3 infection. We found that DRESs in the 3′-UTR region rewired miRNA–mRNA interactions, therefore changing the expression of genes involved in the innate immune response to DHAV-3 infection. In addition, DRESs in the CDS region altered codons of several proteins related to virus infection. These results together provide potentially valuable insights into the role of RNA editing in the susceptibility of ducklings to DHAV-3 infection, which may help the duck industry fight against this disease.

## Data Availability

Datasets used in this study can be obtained from the corresponding authors upon reasonable request.

## Supplementary Materials

The following supporting information can be downloaded at: https://www.mdpi.com/article/10.3390/ijms251910413/s1.
