# Supplementary material for: Genomic Landscape and Regulation of RNA Editing in Pekin Ducks Susceptible to Duck Hepatitis A Virus Genotype 3 Infection
Source: Int J Mol Sci. 2024 Sep 27;25(19):10413. doi: 10.3390/ijms251910413 (PMC11476845; doi:10.3390/ijms251910413)
Supplement: Supplementary file 1 [file ijms-25-10413-s001.zip › ijms-3206160-supplementary.pdf]

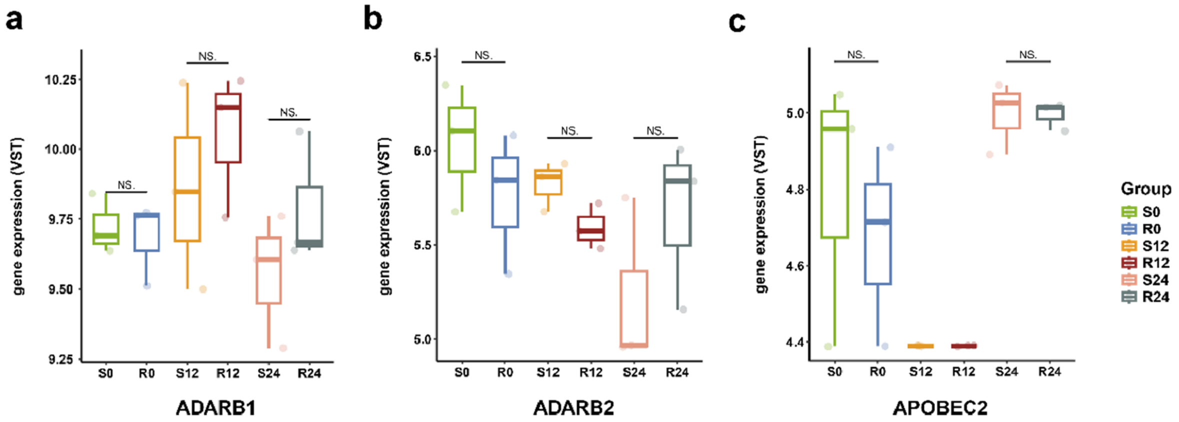

Figure S1. Expression level of ADARB1, ADARB2, and APOBEC2. (a) The expression level of ADARB1 across the DHAV-3 infection in two cultivars. **(b)** The expression level of ADARB2 across the DHAV-3 infection in two cultivars. **(c)** The expression level of APOBEC2 across the DHAV-3 infection in two cultivars.

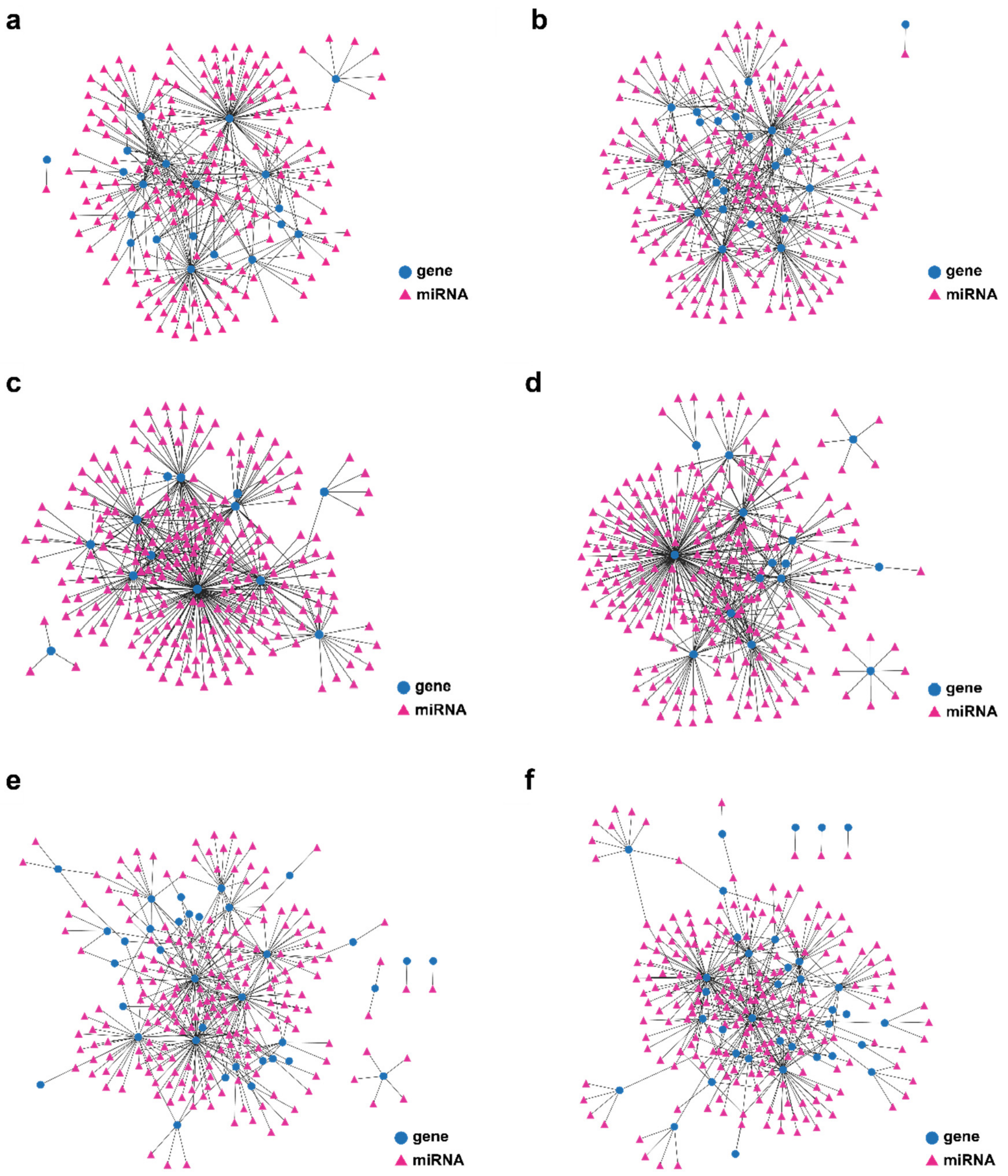

Figure S2 The interaction network of miRNAs and target genes covering DRESs during DHAV-3 infection. (a) The interaction network of miRNAs and wildtype target genes covering DRESs at 0 hpi. **(b)** The interaction network of miRNAs and edited target genes covering DRESs at 0 hpi. **(c)** The interaction network of miRNAs and wildtype target genes covering DRESs at 12 hpi. **(d)** The interaction network of miRNAs and target edited genes covering DRESs at 12 hpi. **(e)** The interaction network of miRNAs and wildtype target genes covering DRESs at 24 hpi. **(f)** The interaction network of miRNAs and edited target genes covering DRESs at 24 hpi.
